# AISleep: Automated and interpretable sleep staging from single-channel EEG data

**DOI:** 10.1016/j.patter.2025.101367

**Published:** 2025-09-24

**Authors:** Xun Mai, Binghua Song, Manli Luo, Jun Zhu, Xu Jiang, Xiao Ma, Feng Lin, Xiaoqing Hu, Hanchuan Peng, Li Zhang, Yina Wei

**Affiliations:** 1 Research Center for Frontier Fundamental Studies, Zhejiang Lab, Hangzhou, China; 2 Institute for Brain and Intelligence, Southeast University, Nanjing, China; 3 Department of Geriatric Neurology, Nanjing Brain Hospital Affiliated to Nanjing Medical University, Nanjing, China; 4 Fuyao University of Science and Technology, Fujian, China; 5 Department of Psychology, The State Key Laboratory of Brain and Cognitive Sciences, The University of Hong Kong, Hong Kong, China; 6 The University of Hong Kong-Shenzhen Institute of Research and Innovation, Shenzhen, China; 7 New Cornerstone Science Laboratory, Institute for Brain and Intelligence, Fudan University, Shanghai, China; 8 Shanghai Academy of Natural Sciences (SANS), Fudan University, Shanghai, China; 9 Department of Biomedical Engineering, Zhejiang University, Hangzhou, China

**Keywords:** sleep, single-channel EEG, power spectral density, PSD, kernel density estimation, KDE, uniform manifold approximation and projection, UMAP, sleep staging, unsupervised algorithm

## Abstract

Sleep staging is essential for understanding sleep physiology and diagnosing sleep-related disorders. However, traditional manual scoring is time-consuming and resource intensive, limiting its scalability for large-scale application. In this study, we introduce AISleep, an automated and interpretable unsupervised algorithm based on feature-weighted kernel density estimation (KDE), designed to stage sleep using only a single electroencephalogram (EEG) channel. AISleep was evaluated using both public benchmark datasets of healthy subjects and clinical datasets of patients with sleep disorders. It outperforms state-of-the-art (SOTA) unsupervised sleep staging algorithms in young, healthy subjects and demonstrates better generalizability compared to supervised models. Importantly, we observed that some key EEG features decline with age, which may contribute to reduced staging accuracy in older adults. This study presents a robust and interpretable unsupervised sleep staging algorithm with a lightweight design that makes it well suited to integration into portable devices, offering a practical and scalable solution for accurate, home-based sleep monitoring.

## Highlights

• AISleep, an unsupervised sleep staging algorithm using single-channel EEG, is presented
• AISleep outperforms SOTA unsupervised and generalizes better than supervised algorithms
• Aging-related declines in EEG features explain reduced staging accuracy in elders

## The bigger picture

We spend nearly a third of our lives asleep, and sleep is fundamental to human health, affecting cognition, metabolism, and overall well-being. Sleep staging, the process of categorizing sleep into distinct phases, is critical for diagnosing sleep disorders and advancing sleep science. However, conventional manual sleep scoring is labor intensive, costly, and impractical for widespread use, limiting opportunities for large-scale sleep monitoring and personalized healthcare. Our work introduces AISleep, an algorithm that requires only one type of signal, single-channel physiological signals, to visualize sleep staging throughout the night. It opens up new possibilities for home-based and personalized sleep health monitoring. By enabling accurate sleep staging outside clinical settings, this technology supports the democratization of sleep assessment and could ultimately reshape how we study sleep, diagnose disorders, and manage long-term health.

## Teaser

Sleep staging is vital for understanding sleep physiology and diagnosing sleep disorders, yet manual scoring is time-consuming and unscalable. The authors introduce AISleep, an unsupervised sleep staging algorithm based on feature-weighted kernel density estimation, using only a single EEG channel. AISleep outperforms SOTA unsupervised methods, generalizes better than supervised models, and uncovers age-related EEG feature decline impacting staging accuracy. Its lightweight design enables integration into portable devices, providing a practical, scalable solution for accurate and accessible home-based sleep monitoring.

## Introduction

Sleep occupies approximately one-third of human life. Identification of sleep stages is fundamental and essential for investigating the sleep physiology underlying cognitive processes such as memory consolidation^1^ and for the diagnosis of several sleep disorders, including narcolepsy,^2^ chronic fatigue syndrome,^3^ insomnia,^4^ and obstructive sleep apnea.^5^ Sleep staging guidelines were first proposed by Rechtschaffen and Kales (R&K)^6^ in 1968, were updated by the American Academy of Sleep Medicine (AASM) in 2007,^7^ and now serve as the current sleep staging criteria. The AASM rules categorize sleep into five stages: Wake, three non-rapid eye movement (NREM) sleep stages (N1, N2, and N3), and rapid eye movement (REM) sleep. Typically, various physiological signals—such as electroencephalogram (EEG), electrooculogram (EOG), electrocardiogram (ECG), and electromyogram (EMG)—are measured during polysomnography (PSG) to determine sleep stages. Among these signals, the EEG signal is the most important, given its unique characteristics in each sleep stage.

Currently, sleep staging is typically performed manually by experts, taking about 2 h to score an overnight PSG recording,^8^ which is both time and resource intensive. In recent years, several automatic sleep staging models using machine learning or deep learning techniques have been proposed, such as DeepSleepNet,^9^ TinySleepNet,^10^ XSleepNet,^11^ U-Sleep,^12^ YASA,^13^ and SleePyCo.^14^ Among these methods, DeepSleepNet^9^ is a seminal deep learning method that combines a convolutional neural network (CNN)^15^ and a long short-term memory (LSTM) network^16^ for sleep staging tasks based on raw single-channel EEG. TinySleepNet,^10^ an improved version of DeepSleepNet consisting of fewer parameters, achieved a similar performance but with less computational cost. XSleepNet^11^ employs two network backbones: one for processing raw EEG signals and another for handling corresponding time-frequency maps, resulting in superior performance compared to TinySleepNet. U-Sleep^12^ is a fully convolutional neural network, trained and evaluated on PSG recordings from 15,660 participants of 16 clinical studies. In contrast to other automated algorithms, it was trained to work with any standard EEG and EOG channels as input. YASA^13^ was proposed after fitting the model with a LightGBM classifier^17^ on 30,000+ h of polysomnographic sleep recordings across heterogeneous populations around the world. This approach demonstrates superior generalization capability compared to deep learning models, establishing YASA as a widely adopted tool for automated sleep staging. Recent work, such as SleePyCo^14^ and EfficientSleepNet,^18^ validates that single-channel EEG remains highly effective for automated sleep scoring. Despite advancements, automatic sleep staging algorithms still face several challenges: the requirement for extensive data to train robust models, variations across different sleep datasets that affect generalizability, the economic burden of data annotation, and the issue of label noise, where there is an approximately 80%–90% moderate agreement rate among experts annotating sleep stages.^19^^,^^20^^,^^21^

In contrast to supervised learning, recent studies have begun to delve into the realm of unsupervised sleep staging algorithms,^22^^,^^23^ which does not depend on prelabeled training datasets. In a pioneering study, Yu et al.^23^ extracted six distinctive features from the frequency domain of one EEG channel and then leveraged an improved *k*-means algorithm with considerations of density and distance metrics to effectively determine the sleep stages. This innovative approach achieved an average accuracy of around 73.5% across six subjects, showcasing the efficacy of their approach in accurately classifying sleep stages without relying on initial training datasets. Subsequently, Decat et al.^22^ extracted over 7,700 features to comprehensively characterize sleep time series using hctsa^24^ (highly comparative time-series analysis) and then applied the *k*-means clustering algorithm to categorize the sleep data into the five standard sleep stages, leading to average recalls of around 61%, 53.3%, 43.1%, 77.4%, and 60.2% for Wake, N1, N2, N3, and REM. Interestingly, despite variations in algorithms and sleep datasets, the feature embeddings of sleep stages, whether encoded by diverse deep learning architectures^25^^,^^26^ or extracted directly from time-series EEG/EMG data,^22^^,^^27^ demonstrate a consistent manifold pattern. This pattern corresponds to transitions between distinct sleep stages, indicating a structured representation of stage changes within the sleep cycle.

Inspired by this consistent manifold pattern of sleep transitions, we propose AISleep, an innovative unsupervised algorithm for sleep staging, which utilizes uniform manifold approximation and projection (UMAP)^28^ for dimensionality reduction on the power spectral density (PSD) of a frontal single-channel EEG recording and then employs feature-weighted kernel density estimation (KDE)^29^^,^^30^ to infer the distributions of different sleep stages. We have found that AISleep can visually show the distribution of and variation in sleep states throughout the entire night, providing reliable sleep staging. To validate the accuracy and robustness of AISleep, it was evaluated on public datasets (SleepEDF^31^^,^^32^) and a private clinical sleep disorder dataset (NJ-EDF). We found that AISleep achieved superior accuracy in the unsupervised sleep staging algorithms and outperformed supervised deep learning models on the unseen datasets while also being much more robust and interpretable. This study demonstrates the feasibility of using unsupervised techniques for sleep staging without the need for human experts, which might be particularly suitable for use with portable devices in home environments.

## Results

### Identification of the open-eye Wake state

**Figure 1:**
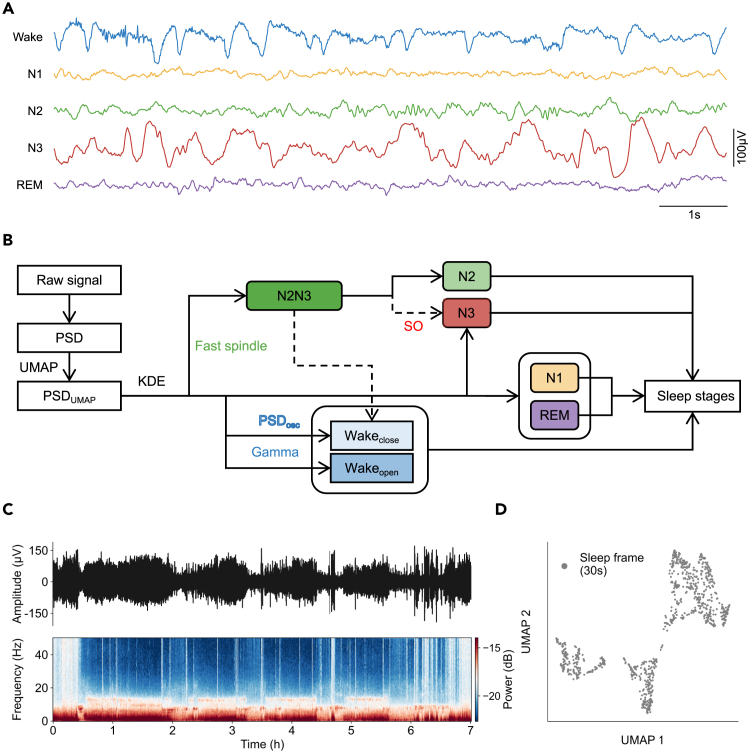
The AISleep workflow (A) Representative EEG traces across different sleep stages: Wake, N1, N2, N3, and REM. (B) Overview of the AISleep pipeline. For each 30-s single-channel EEG epoch from overnight recordings, we computed the power spectral density (PSD). These high-dimensional PSD features within the 0.2- to 30-Hz frequency range were then projected into a two-dimensional space using UMAP (uniform manifold approximation and projection), yielding PSD_UMAP_. The characteristic signals, such as fast spindle, slow oscillations (SO), gamma power, and PSD_osc_ (the oscillatory component of PSD), were used in combination with kernel density estimation (KDE) to identify clusters corresponding to distinct sleep stages. (C) Top: a representative EEG recording during an overnight sleep (subject SC4001, channel Fpz-Cz) from the SleepEDF-20 dataset. Bottom: the corresponding spectrogram computed by calculating the PSD of non-overlapping 30-s EEG epochs throughout the night. (D) PSD_UMAP_ visualization of all 30-s EEG epochs across an entire night. Each point represents the PSD_UMAP_ corresponding to a single sleep frame (30-s EEG epoch).

During sleep, although the brain is decoupled from sensory input, the EEG signals exhibit distinct patterns of activity across the different sleep stages: Wake, N1, N2, N3, and REM (Figure 1A; see Table S1 for a brief overview of characteristic features in each stage). These EEG patterns are key features for accurate sleep staging. In this study, we followed the workflow of AISleep to identify different sleep stages (Figure 1B) for each one-night sleep recording. We first calculated the PSD for each 30-s EEG epoch (denoted as a sleep frame) from the Fpz-Cz channel across the entire night (Figure 1C). To intuitively understand the feature distribution of each sleep stage during a one-night sleep, we utilized UMAP to map the high-dimensional PSD (within the frequency range of 0.2–30 Hz) into a two-dimensional PSD_UMAP_ (Figures 1D and S1). Embeddings (PSD_UMAP_) associated with identical sleep stages typically exhibit a clustering pattern, while embeddings from different sleep stages are generally found to be segregated from one another. To identify each 30-s sleep frame corresponding to a specific sleep stage, we used KDE^29^^,^^30^ to estimate the probability density function based on PSD_UMAP_.

**Figure 2:**
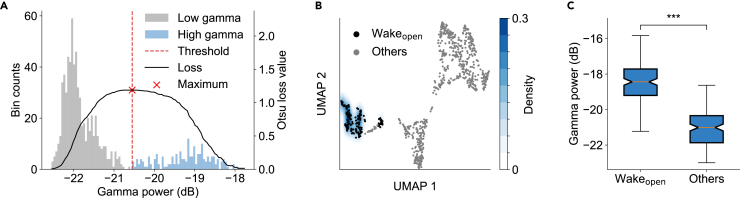
Identification of the open-eye Wake state (A) Distribution of gamma power for every 30-s sleep frame across the night (subject SC4001, channel Fpz-Cz). The Otsu method was applied to segment the bimodal distribution (gray, low gamma; light blue, high gamma), with the threshold (red dotted line) corresponding to the gamma power value that maximizes (red cross) the between-class variance (Otsu loss function, black line). (B) Probability density of the open-eye Wake (Wake_open_) state estimated by KDE. The blue region highlights areas associated with higher gamma power, where color intensity reflects KDE density. Black points denote individual sleep frames identified as Wake_open_. (C) Comparison of the average gamma power between the Wake_open_ stage and other sleep stages among all recorded nights from SleepEDF-78 (*n* = 153 nights, paired t test, two-tailed, *p* = 2.46 × 10^−99^). Boxplots show the median (center line), first and third quartiles (box limits), and whiskers extending to 1.5× the interquartile range (IQR). ∗∗∗*p* < 0.001.

The characteristic features of the Wake stage depend on whether the eyes are open or closed. During open-eye Wake state (Wake_open_), EEG typically exhibits low-amplitude, high-frequency activity at gamma frequencies, which is associated with attention and memory.^33^ Taking one night of sleep recording as an example (subject SC4001, channel Fpz-Cz), the distribution of the gamma power of every 30-s sleep frame indicated the presence of two distinct distributions: that with lower gamma power and that with higher gamma power (Figure 2A). Sleep frames exhibiting higher gamma power predominantly corresponded to the Wake_open_ state. To differentiate between high and low gamma power, we used the Otsu algorithm^34^ to automatically determine the threshold (Figure 2A, red dashed line).

In the PSD_UMAP_ plane during one night of sleep (Figure 2B, gray dots), we calculated the probability density p for each point using KDE, with the weights of KDE determined by the gamma power (see methods). For the region where the density p exceeds 10% of the global density maximum $p_{\max}$, we denoted it in blue. We then identified the sleep frames within blue regions as Wake_open_ (Figure 2B, black dots). Among all the recorded subjects (*n* = 153 nights), we found that the average gamma power during the Wake_open_ state was significantly higher compared to other sleep stages (Figure 2C, *p* < 0.001).

### Identification of the N2N3 stage

**Figure 3:**
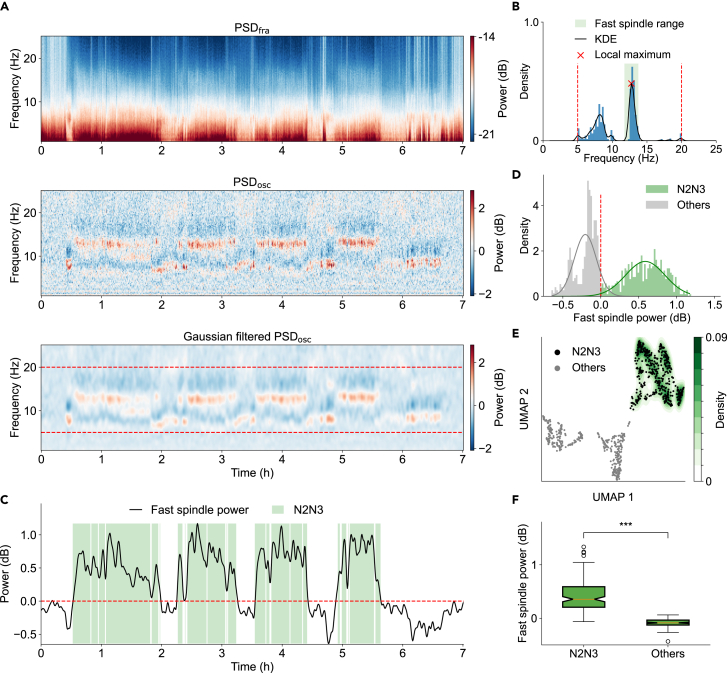
Identification of the N2N3 stage (A) An example of PSD decomposition during one night of sleep (subject SC4001, channel Fpz-Cz). Top: PSD_fra_, the fractal component extracted using the irregular resampling auto-spectral analysis (IRASA) algorithm. Middle: PSD_osc_, the oscillatory component obtained by subtracting PSD_fra_ from PSD. Bottom: Gaussian-filtered PSD_osc_. (B) Histogram of the peak ${\text{PSD}}_{\text{osc}}$ frequency (blue line) in the range of 5–20 Hz (red dotted lines) during whole-night sleep. The smoothed probability density distribution was estimated using KDE (black line). The central frequency was defined as the local maximum (red cross) in the vicinity of the fast spindle range (12–16 Hz). The personalized fast spindle range was defined as the central frequency ± 1 Hz (light green region). (C and D) The dynamics (C, dark line) and the histogram (D) of the personalized fast spindle power ($P_{sp}$) throughout the night. The distribution of the personalized $P_{sp}$ can be divided into two groups based on the threshold at zero (dotted red line). The probability density distributions of two groups were estimated using Gaussian distributions (D, light gray line and light green line, respectively). The green region corresponds to the N2N3 stage identified by AISleep. (E) The probability density of the N2N3 stage estimated by KDE. The green region corresponds to areas with elevated *P*_*sp*_, where color intensity reflects KDE density. Black points correspond to the sleep frame identified as the N2N3 stage. (F) Comparison of the personalized $P_{sp}$ between the N2N3 stage and other stages among all recorded nights from SleepEDF-78 (*n* = 153, Wilcoxon signed-rank test, *p* = 7.39 × 10^−27^). Boxplots show the median (center line), first and third quartiles (box limits), and whiskers extending to 1.5× IQR. ∗∗∗*p* < 0.001.

To accurately identify the N2N3 stage, characterized by spindle activities that primarily encompass both the N2 and significant portions of the N3 stage, we first separated the fractal (1/f brain activities) and oscillatory (primarily including spindles and alpha waves observed during sleep) components in the power spectrum. We utilized the irregular resampling auto-spectral analysis (IRASA) method^35^ to decompose the PSD (for example, Figure 1C, bottom) of each sleep frame into PSD_fra_ (Figure 3A, top) and PSD_osc_ (Figure 3A, middle). This approach allowed us to specifically focus on the oscillatory components of interest, facilitating their clear visualization and further analysis.

We then identified the peak frequency corresponding to the peak of the Gaussian-filtered PSD_osc_ (Figure 3A, bottom) in the range of 5–20 Hz. The peak frequency during one night of sleep showed a bimodal distribution (Figure 3B), indicating distinct alpha and spindle activity. The personalized fast spindle power ($P_{sp}$), calculated around the peak frequency closest to 14 Hz (Figure 3B, red marker “×”) for each sleep frame, varied throughout the night (Figure 3C, dark line). The distribution of the personalized $P_{sp}$ also demonstrated a bimodal distribution (Figure 3D), representing high spindle power (Figure 3D, green) and low spindle power (Figure 3D, gray). This bimodal distribution highlights the fact that spindle activities vary in amplitude across the night, possibly reflecting distinct stages of sleep.

We then identified the N2N3 stage based on the probability density estimated from the KDE with weights related to the personalized spindle power (Figure 3E, see methods) during one night of sleep. Across all recorded subjects and nights, we observed that the personalized $P_{sp}$ in the N2N3 stage was significantly higher than in the other stages (Figure 3F, *p* < 0.001). These findings provide valuable insights into the distinctive characteristics of spindle activities during the N2N3 stage of sleep.

### Identification of the N3 stage

**Figure 4:**
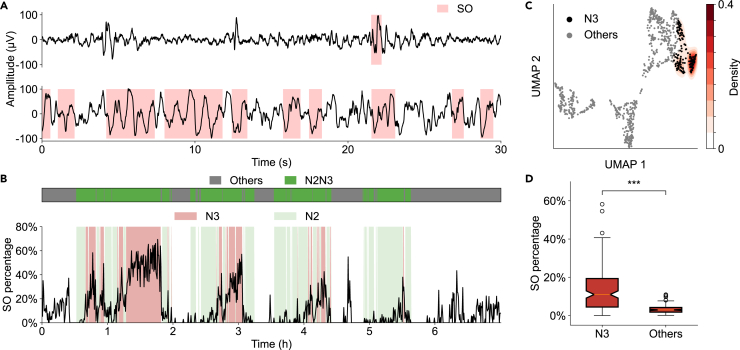
Identification of the N3 stage (A) Two representative examples of detected slow oscillations (SOs). Top: low SO percentage (2.3%). Bottom: high SO percentage (49.6%). Detected SOs are highlighted in red. (B) Temporal dynamics of SO percentage across the whole night (dark line). Red regions represent the N3 stage. Light green regions represent the N2 stage. Dark green regions represent the N2N3 region. (C) Probability density of the N3 stage estimated by KDE. The red area in the PSD_UMAP_ represents a higher SO percentage, where color intensity reflects KDE density. (D) Comparison of the average SO percentage between the N3 stage and all other stages among all SleepEDF-78 recordings (*n* = 153, Wilcoxon signed-rank test, *p* = 1.78 × 10^−25^). Boxplots show the median (center line), first and third quartiles (box limits), and whiskers extending to 1.5× IQR. ∗∗∗*p* < 0.001.

The N3 stage is characterized by the presence of slow oscillation (SO) activities, which are a hallmark of this stage. By detecting the SOs (Figure 4A, light red regions) and calculating the percentage of SOs present in each sleep frame, we found that the proportion of SOs fluctuated throughout the entire duration of sleep (Figure 4B, black line). Noisy signals, such as eye movements and body movements, might be misinterpreted as SOs. We excluded the effect of noisy signals and the detected Wake_open_ stage (see methods), and we calculated the probability density of KDE based on the SO percentages that defined the region of the N3 stage (Figure 4C, red). The SO percentage rose predominantly during the N3 stage (Figure 4B, light red). The rest of the N2N3 region would be determined as the N2 stage (Figure 4B, light green). Among all recorded nights from SleepEDF-78, we found that the mean of the SO percentage showed a significant difference during N3 sleep compared to other sleep stages (Figure 4D, *p* < 0.001).

### Identification of the closed-eye Wake state

**Figure 5:**
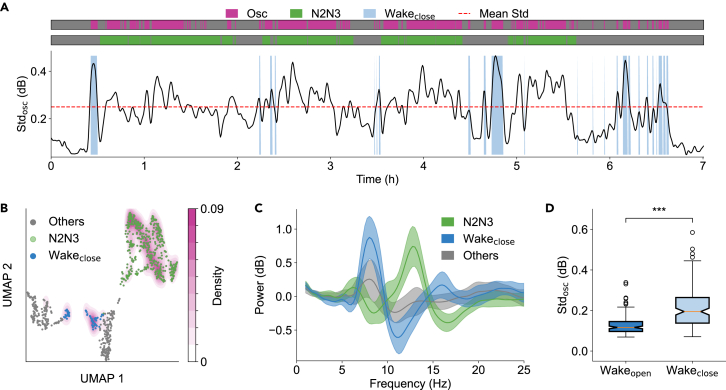
Identification of the Wake_close_ state (A) Temporal dynamics of oscillatory activity throughout the whole night (subject SC4001). For each 30-s sleep frame, the oscillatory standard deviation (${\text{Std}}_{\text{osc}}$) was computed as the standard deviation of Gaussian-filtered PSD_osc_ in the 5–20 Hz range. The dotted red line represents the mean ${\text{Std}}_{\text{osc}}$ across the entire night. The purple-red, green, and light green regions represent the oscillatory states, the N2N3 state, and the Wake_close_ state, respectively. (B) Density distribution of high oscillatory activity estimated by KDE. The oscillatory states (purple-red) include the large amount of N2N3 (green), characterized by high spindle activities, and Wake_close_ (light blue), characterized by high alpha activities. The color intensity reflects KDE density. (C) The PSD_osc_ in the Wake_close_, N2N3, and other stages. The error bands represent the mean ± standard deviation of PSD_osc_. (D) Comparison of mean ${\text{Std}}_{\text{osc}}$ between the Wake_open_ and the Wake_close_ states among all recorded nights from SleepEDF-78 exhibiting alpha oscillations (*n* = 139, Wilcoxon signed-rank test, *p* = 4.78 × 10^−20^). Boxplots show the median (center line), first and third quartiles (box limits), and whiskers extending to 1.5× IQR. Note: approximately 10% of subjects did not exhibit closed-eye alpha rhythms, resulting in *n* = 139 rather than *n* = 153 for the comparison. ∗∗∗*p* < 0.001.

To identify the closed-eye Wake state (Wake_close_), we first needed to determine whether the sleep frame had the oscillatory activities within the frequency range of 5–20 Hz, primarily exhibiting spindle and alpha activities. We quantified the strength of oscillatory power using the standard deviation of Gaussian-filtered PSD_osc_ (frequency range: 5–20 Hz), which varied throughout the night (Figure 5A, black line). We estimated the probability density of KDE based on the standard deviation of PSD_osc_ to identify the oscillatory region (Figure 5B, purple-red) characterized by spindle and alpha activities. In the sleep frames exhibiting oscillatory activities, we excluded those identified as the N2N3 stage (Figure 5B, green), where oscillatory power originates from spindle activity. The remaining sleep frames, which exhibited alpha activities, were identified as the ${\text{Wake}}_{\text{close}}$ state (Figure 5B, blue).

By comparing the PSD_osc_ of sleep frames during the Wake_close_ state, the N2N3 stage, and other sleep stages (Figure 5C), we observed distinct patterns in the power distribution. Specifically, the Wake_close_ state exhibited a prominent power in the alpha band (Figure 5C, blue), while the N2N3 stage demonstrated a strong power in the spindle range (Figure 5C, green). These findings suggest distinct neural oscillations associated with different sleep stages. Among all recorded nights from the SleepEDF-78 dataset, the standard deviation of PSD_osc_ in the Wake_close_ state was significantly higher than that of the Wake_open_ state (Figure 5D).

Building upon our identification of Wake_open_ (Figure 2B) and Wake_close_ (Figure 5B), their union constitutes the Wake stage. Intriguingly, sleep frames in the intersection of Wake_open_ and Wake_close_ exhibited mixed gamma and alpha activity (Wake_overlap_, Figure S2, brown). Both PSD_UMAP_ embedding (Figure S2B) and spectral analysis (Figures S2C–S2E) consistently revealed that Wake_overlap_ represents a transitional state between Wake_open_ and Wake_close_, indicating that sleep stages exist on a continuum.

### Estimation of the N1 and REM stages

**Figure 6:**
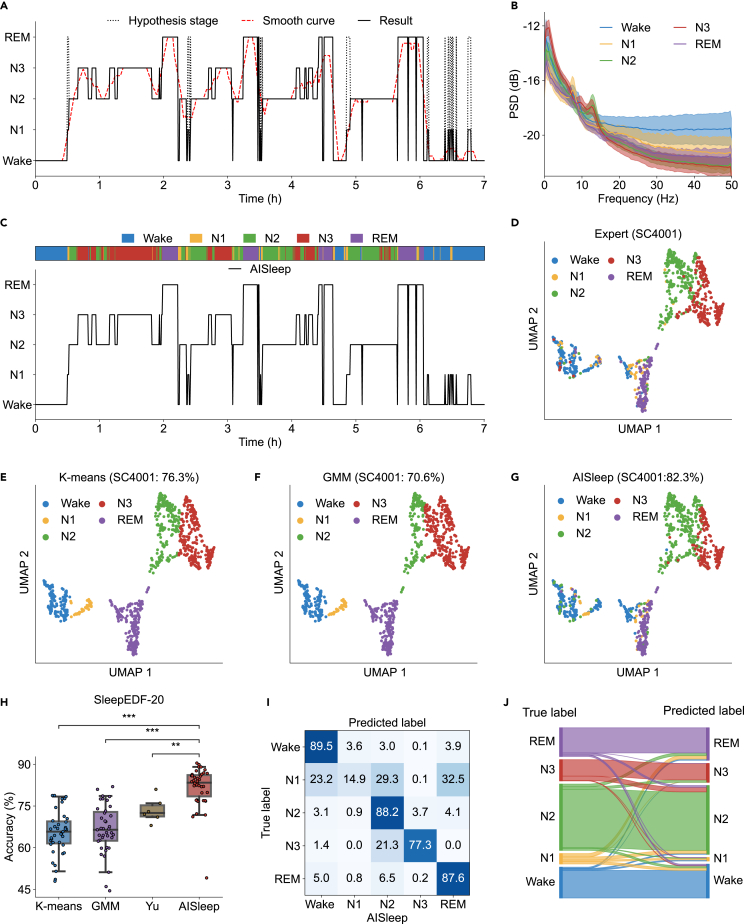
Comparison of AISleep with unsupervised algorithms (A) Estimation of N1 and REM stages (subject SC4001). The dotted black line represents the hypothesized curve, the dotted red line represents the smoothed curve, and the solid black line represents the adjusted stage results. (B) Differences in PSD among sleep stages estimated by AISleep. The error bands represent the mean ± standard deviation of PSD for each stage. (C) Comparison of AISleep (black line) with human-annotated sleep stages (top colored region). (D–G) Low-dimensional embeddings PSD_UMAP_, color-coded according to the sleep stages from human annotation (D), *k*-means (E), Gaussian mixture model (GMM; F), and AISleep (G) for subject SC4001. (H) Comparison of AISleep with other unsupervised methods (*k*-means, GMM, and Yu et al.’s method^23^) on the SleepEDF-20 dataset. Boxplots show the median (center line), first and third quartiles (box limits), and whiskers extending to 1.5× IQR. Statistical significance was assessed using one-way ANOVA with Holm correction for multiple comparisons. Each dot represents one subject. ∗∗∗*p* < 0.001 and ∗∗*p* < 0.01. (I and J) Normalized confusion matrices (I) and Sankey diagram (J) illustrating the performance of AISleep on the SleepEDF-20 dataset.

After identifying the sleep frames of the Wake, N2, and N3 stages, we attributed the remaining unknown frames predominantly to the N1 and REM stages. We provisionally labeled these frames as the hypothesized REM stage (Figure 6A, dotted line) and dynamically adjusted the hypothesized REM stage to the N1 stage based on the smoothed curve (Figure 6A, dotted red) by incorporating the sleep transition patterns, resulting in the final determination of sleep stages (Figure 6A, black line, see methods). The mean PSD of each sleep stage from the AISleep algorithm (Figure 6B) showed that gamma power was notably higher during the Wake stage than in other sleep stages. Additionally, pronounced peaks in PSD were observed within the spindle frequency range for the N2 and N3 stages, with the N3 stage showing a particularly strong presence in the delta (0–4 Hz) frequency band (Figure 6B).

The sleep stages identified by the AISleep algorithm (Figure 6C, black line) showed strong agreement with expert manual scoring (Figure 6C, top colored box), with optimal performance achieved at a two-dimensional UMAP embedding (Figure S3). From the distribution of PSD_UMAP_ in different sleep stages (Figure 6D), we found that the Wake stage was closer to the N1 and REM stages. By contrast, the N2 stage was situated intermediately between the N3 and the N1/REM stages. This indicates that the relative positions of sleep stages on the PSD_UMAP_ correspond to the transitions between sleep states. These results suggest that the AISleep algorithm effectively and precisely classifies sleep frames into their appropriate stages.

### Comparison between AISleep and other unsupervised algorithms

To assess the effectiveness of unsupervised sleep staging algorithms, we compared AISleep with two traditional clustering algorithms, *k*-means and Gaussian mixture model (GMM) using the PSD_UMAP_ features extracted from single-channel EEG recordings in the SleepEDF-20 dataset.^31^^,^^32^ Compared with human annotated sleep stages (Figure 6D), traditional clustering algorithms (Figures 6E and 6F) showed a considerable degree of similarity, while AISleep (Figure 6G) demonstrated significantly superior performance over both *k*-means and GMM on the SleepEDF-20 dataset (Figure 6H). These findings highlight the effectiveness of combining UMAP with feature-weighted KDE synergistically to enhance unsupervised clustering techniques.

In addition, we compared the performance of AISleep with that of unsupervised sleep staging algorithms based on an improved *k*-means algorithm proposed by Yu et al.^23^ While Yu et al. evaluated their method on six subjects from the SleepEDF-20 dataset, we tested *k*-means, GMM, and AISleep on all 20 subjects. Our results showed that AISleep significantly outperformed Yu’s approach (Figure 6H, Mann-Whitney U test, *p* = 0.0089), with an improvement of approximately 8.5% (82.0% ± 5.4% vs. 73.5% ± 4.1%). Furthermore, AISleep exhibited consistently high performance across all sleep stages, except for N1, which typically constitutes a relatively small proportion of sleep (Figures 6I and 6J). Among the unsupervised algorithms evaluated, AISleep emerged as the most effective method for sleep staging using only a single EEG channel.

### Comparison between AISleep and supervised algorithms

Automated sleep staging using data-driven supervised models typically involves training on large labeled sleep datasets and evaluating on subjects from the same data distribution. However, in practical applications, the training and test datasets often come from different distributions. To evaluate on an unseen dataset, we compared the performance of supervised models in cross-domain testing with that of AISleep on healthy subjects, including an elderly population (SleepEDF-78) and patients with sleep disorders (NJ-EDF), as summarized in Table 1.

**Table 1:** Evaluation of sleep staging algorithms on the unseen dataset

Method	Train	Test	Overall metrics			Per-class F1 score (F1)				
			Acc	MF1	κ	W	N1	N2	N3	REM
YASA	Others dataset	SleepEDF-78 (*n* = 153)	70.6	60.1	58.1	77.2	17.1	76.7	64.8	64.7
TinySleepNet	NJ-EDF		50.4	45.4	32.0	53.7	**19.6**	56.4	54.0	43.5
SleePyCo	NJ-EDF		53.3	46.1	36.8	58.3	12.8	58.6	51.6	49
**AISleep (ours)**	**–**		**76.1**	**65.7**	**66.4**	**86.0**	17.5	**80.2**	**71.0**	**73.7**
YASA	Others dataset	NJ-EDF (*n* = 42)	59.6	41.5	40.0	71.9	10.5	62.9	19.3	42.8
TinySleepNet	SleepEDF-78		61.2	51.9	44.9	74.1	**25.0**	63.3	52.7	44.2
SleePyCo	SleepEDF-78		63.2	**54.5**	48.7	77.9	23.6	**66.7**	58.8	**45.4**
**AISleep (ours)**	**–**		**66.0**	53.4	**52.1**	**82.5**	18.5	64.3	**60.6**	40.9

The overall metrics include accuracy (Acc), macro F1 score (MF1), Cohen’s kappa (κ), and per-class F1 scores, with all values reported as percentages.

**Figure 7:**
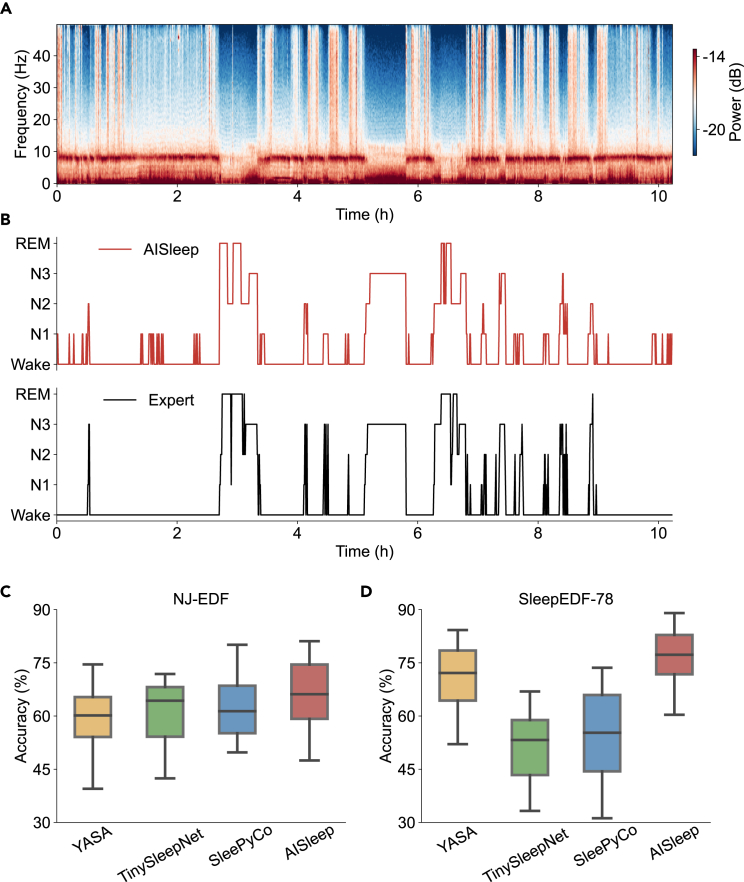
Comparison of AISleep with supervised sleep staging algorithms (A) The spectrogram of a one-night EEG recording from a sleep disorder patient in NJ-EDF (channel F3-M2). (B) Sleep staging scored by AISleep and human experts. (C and D) Comparison of sleep staging algorithms under cross-domain testing using the NJ-EDF (C) and SleepEDF-78 (D) datasets. Boxplots show the median (center line), first and third quartiles (box limits), and whiskers extending to 1.5× IQR.

When evaluating algorithm performance on the NJ-EDF dataset (Figure 7), we observed notable differences in sleep patterns between patients and healthy subjects, as evidenced by EEG spectrograms (Figure 7A) and expert annotations (Figure 7B, bottom). Although AISleep, YASA,^13^ TinySleepNet,^10^ and SleePyCo^14^ all showed reduced performance in sleep disorder patients compared to healthy subjects, AISleep maintained the highest accuracy among all algorithms (Figure 7C; Table 1). Furthermore, in healthy subjects from the SleepEDF-78 dataset, AISleep outperformed YASA, TinySleepNet, and SleePyCo during cross-domain testing (Figure 7D), demonstrating its superior generalizability to unseen data.

These results collectively indicate that, while supervised models like YASA, TinySleepNet, and SleePyCo experienced significant performance declines under cross-domain testing conditions, AISleep, as an unsupervised algorithm, maintained consistent efficiency and robustness across unseen datasets. This highlights its potential advantage in real-world scenarios where training and test data distributions may differ.

### The effect of age on sleep staging

**Figure 8:**
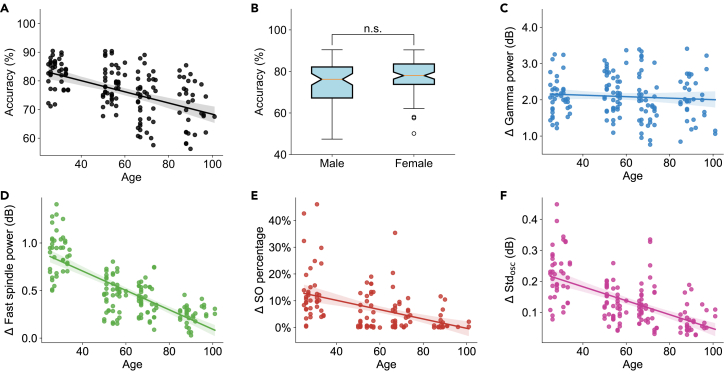
The impact of age and gender on the performance of the AISleep algorithm (A) The relationship between age and the performance of AISleep (R^2^ = 0.191; Spearman’s correlation coefficient ρ = −0.47; *p* = 6.69 × 10^−8^). (B) The performance of AISleep between males (*n* = 71) and females (*n* = 82) (Mann-Whitney U test, *p* > 0.05). Boxplots show the median (center line), first and third quartiles (box limits), and whiskers extending to 1.5× IQR. n.s. indicates no significance. (C) The relationship between age and Δ gamma power (R^2^ = 0.006; Spearman’s ρ = −0.11; *p* = 0.16). Δ gamma power was computed as the difference between the average gamma power during the Wake_open_ stage and that of other sleep stages. (D) The relationship between age and Δ fast spindle power (R^2^ = 0.581; Spearman’s ρ = −0.72; *p* = 1.28 × 10^−25^). Δ fast spindle power was computed as the subtraction of the mean fast spindle power during the N2N3 stages from that of other sleep stages. (E) The relationship between age and Δ SO percentage (R^2^ = 0.191; Spearman’s ρ = −0.47; *p* = 6.69 × 10^−8^). Δ SO percentage was computed as the subtraction of the SO percentage in the N3 stage from that of other sleep stages. (F) The relationship between age and ${\Delta \text{Std}}_{\text{osc}}$ (R^2^ = 0.361; Spearman’s ρ = −0.61; *p* = 9.37 × 10^−17^). ${\Delta \text{Std}}_{\text{osc}}$ was computed as the difference between the average ${\;\text{Std}}_{\text{osc}}$ above and below the whole-night mean (dotted red line in Figure 5A). The shaded area represents the 95% confidence interval for the regression estimate.

We observed that the accuracy of the AISleep algorithm decreases with increasing age (Figure 8A). However, no significant differences in accuracy were observed between male and female groups (Figure 8B). Additionally, existing sleep staging algorithms also exhibited lower accuracy with the SleepEDF-78 dataset compared to the SleepEDF-20 dataset. The difference in accuracy may stem from differences in the age compositions of the two datasets: the population of SleepEDF-20 is primarily composed of young adults, while the SleepEDF-78 dataset includes a larger proportion of middle-aged and elderly individuals.

To better understand the influence of age on the efficacy of the AISleep algorithm, we explored the relationships between age and four key features, comprising gamma power, *P*_*sp*_, the SO percentage, and the strength of oscillatory activities (${Std}_{osc}$), used in the AISleep algorithm (Figures 8C–8F). Our analysis results indicated that the increase in age did not result in significant alterations in gamma power across the Wake_open_ stage and other stages (Figure 8C). However, the distinction in *P*_*sp*_ between the N2N3 stages and the remaining stages was found to diminish with age (Figure 8D). This observation aligns with previous studies,^36^^,^^37^^,^^38^ which demonstrated an inverse relationship between spindle power and age. Additionally, we noted that both the SO activity (Figure 8E) and the oscillatory activity (Figure 8F) were decreased with age. These findings indicated that, except for gamma power, the distinction of the other three key features becomes less pronounced with age, which could consequently make it more challenging to differentiate between different sleep states in older individuals. This insight provides a plausible explanation for how age factors affect the accuracy of AISleep.

## Discussion

In this study, we propose AISleep, an innovative unsupervised algorithm for sleep staging that eliminates the need for manual annotations and training processes. Our approach leverages a set of key features, including gamma power, *P*_*sp*_, the SO percentage, and the strength of oscillatory activities, to estimate kernel density for PSD_UMAP_ embeddings across various sleep stages. By integrating data-driven clustering with AASM staging guidelines, AISleep not only achieved higher accuracy than state-of-the-art (SOTA) unsupervised sleep staging algorithms, but also offers enhanced robustness and interpretability when compared to supervised algorithms. This study highlights the feasibility of using unsupervised techniques for sleep staging, making it an ideal solution for portable devices in home settings where human expert intervention is minimal or absent.

### The inherent advantages of unsupervised algorithms in AISleep

We have evaluated AISleep on both healthy subject datasets and clinical sleep disorder patient datasets. Compared to existing unsupervised sleep staging algorithms,^22^^,^^23^ AISleep achieved significant performance improvements, with an accuracy rate approximately 8.5% higher than the SOTA unsupervised algorithm.^23^

In terms of feature selection, AISleep employs a more compact feature set compared to Decat’s method.^22^ For unsupervised algorithms, the quality of features is paramount. High-quality features are more valuable than a large number of low-quality ones.^39^ Many time-series features may not correlate with sleep stages and fail to contribute additional insights to the model while unnecessarily increasing the computational complexity.

Regarding algorithmic approaches, although *k*-means and GMM methods are widely used due to their simplicity and effectiveness, they are constrained by issues such as sensitivity to initial centroids and a tendency to produce balanced clusters.^40^ By contrast, AISleep, based on feature-weighted KDE, does not presuppose any specific data distribution and is independent of initial centroids, which endows it with a high degree of flexibility and adaptability to diverse data structures.

### The generalization ability for unseen domains

In most studies on data-driven supervised sleep staging algorithms, the training and testing data are from the same dataset. However, in practical applications, the training and test datasets often come from different distributions. As a result, the performance of these models can be expected to degrade significantly when applied to practical situations.^41^ In the study by Alvarez-Estevez and Rijsman,^42^ they also showed that supervised algorithms often exhibit lower accuracy in cross-domain testing compared to within-dataset cross-validation.

On the unseen datasets, the performance of AISleep and YASA exhibits relatively minor variations on different datasets, with AISleep consistently outperforming YASA. By contrast, the performance of deep learning methods like TinySleepNet and SleePyCo shows greater fluctuations. In cross-domain testing, the accuracy of TinySleepNet and SleePyCo on SleepEDF datasets is significantly lower than the accuracy in their study^10^ of N-fold cross-validation. When the distribution of training data differs from that of the testing data, the model’s cross-domain testing performance is influenced by the model’s parameter count.^43^^,^^44^ The variation of accuracy is related to the degree of model overfitting, as models with a large number of parameters often have a strong fitting capability that increases their susceptibility to overfitting. Various factors could contribute to the lack of homogeneity among different sleep datasets,^45^ including variations in EEG sensors and subjects’ physiological condition, health status, psychological stress, fatigue levels, and sleep environment. Additionally, differences in intrascorer subjectivity^21^ could also introduce further label noise.^46^ Furthermore, the adaptability of algorithms to different datasets remains a concern. The objective of supervised algorithms is to identify a classification hyperplane in high-dimensional feature space that suits all sleep datasets. However, significant distribution differences across datasets may prevent the establishment of an ideal hyperplane, resulting in variations in the performance of supervised methods.

Previous studies revealed that the feature distribution of different sleep stages, whether based on multiple nocturnal sleep records^47^ or a single night,^25^ shows similar sleep stages clustering together with some overlap. This overlap indicates that classifiers encounter challenges in establishing a discriminative boundary. In this study, the selected features are not used directly for clustering but serve as weights to adjust the boundary of the KDE. The probability density function of KDE is also determined by the inherent distribution of the PSD_UMAP_. Therefore, the clustering boundaries in AISleep are jointly determined by the data distribution and the selected features, allowing AISleep to demonstrate strong adaptability and generalization capabilities.

### The potential factors affecting the performance of sleep staging

We have observed a decrease in the accuracy of AISleep with increasing age and a decline in patients with sleep disorders. This trend can be attributed to three primary factors:

(1) Age of subjects: we have observed variations in the performance of AISleep across different age groups. Our experimental data indicate that these age-related fluctuations in performance can be attributed to a consistent decline in EEG features during sleep. Specifically, there is a notable decrease in *P*_*sp*_, the percentage of SOs, and the intensity of oscillatory signals. Previous studies have also shown reductions in SOs and spindle activities associated with aging.^36^^,^^48^ This attenuation of EEG characteristics reduces the distinctiveness between sleep stages, compromising the algorithm’s ability to accurately stage sleep. The subtler differences in EEG patterns with age make it more challenging for AISleep and other algorithms to differentiate between stages, leading to a decrease in accuracy.
(2) Health status of subjects: AISleep demonstrates superior performance on datasets from healthy subjects compared to those with sleep disorders. Patients with sleep disorders exhibited disrupted sleep structures, with sleep stage transitions becoming erratic and losing their normal periodicity.^4^ In such scenarios, where the typical data distribution pattern is altered, the performance of AISleep, YASA, TinySleepNet, and SleePyCo, as shown in our study, tends to deteriorate. The decline in performance was similarly observed in a recent study^49^ where their generalizable sleep staging algorithm was applied to datasets comprising individuals with diverse sleep disorders. To further improve the model’s generalizability and performance in real-world applications, we should include more demographically diverse datasets in our future research.
(3) EEG channel location: the selection of the single channel critically impacts the performance of automatic sleep staging, as specific waveforms characterizing sleep stages are most prominent in particular brain regions. For instance, sleep spindles and SOs are primarily observed in frontal and parietal regions, while alpha rhythms are strongest in the occipital region. To optimize performance, electrode placement should prioritize frontal signal capture while also ensuring sensitivity to occipital alpha activity. In this study, Fpz-Cz (for Sleep-EDF) and F3-M2 (for NJ-EDF) were chosen, both of which feature frontal sampling with a posterior reference electrode. This setup enables high signal-to-noise ratio (SNR) for spindles and SOs, while still preserving sufficient alpha power. Prior studies have shown that Fpz-Cz outperforms Pz-Oz by approximately 2% in Sleep-EDF.^9^ For future studies or applications using a single EEG channel, we recommend F3-M2 or F4-M1, aligning with AASM guidelines. If these options are unavailable, it is recommended to select channels with the sampling electrode positioned near the frontal area and the reference electrode placed near the occipital region to preserve alpha rhythm detectability.

### Unsupervised staging captures gradual age-related changes in sleep patterns

Prior research has established characteristic age-dependent alterations in sleep, including reduced slow-wave sleep^50^ and increased fragmentation.^51^ However, supervised staging approaches, which rely on discrete AASM-defined stages, often overlook subtle but biologically meaningful transitions in sleep microstructure. Our findings demonstrate that unsupervised sleep staging provides unique insights into gradual age-related changes in sleep architecture that are often missed by conventional supervised approaches. Specifically, we identify: (1) declines in *P*_*sp*_, which might contribute to the age-related memory consolidation deficits^52^; (2) reductions in SOs, reflecting diminished cortical synchronization and potential cognitive decline^53^^,^^54^; and (3) attenuation of alpha oscillatory activity, which may reflect fundamental changes in thalamocortical circuits associated with aging.^55^

These continuous, quantifiable metrics offer several advantages over categorical staging: they may better capture transitional sleep states that are clinically meaningful but poorly classified by standard systems and could serve as sensitive biomarkers for age-related neurological changes. The ability to track these gradual alterations has important implications for developing more precise monitoring tools, particularly for aging populations where subtle sleep disturbances often precede cognitive decline. Furthermore, our approach could enhance the personalization of sleep interventions by identifying individual trajectories of age-related sleep deterioration rather than relying on population-wide stage definitions. Our study suggests that unsupervised methods may provide complementary information about sleep’s neurobiological continuum, particularly for research applications investigating the interface between normal aging and early neurodegenerative processes.

### Practical advantages of single-channel EEG for sleep staging

In developing AISleep, we prioritized single-channel EEG-based features to ensure broad applicability in resource-constrained scenarios, such as wearable devices at home-based monitoring. While multimodal signals (e.g., EOG/EMG) or high-density (HD) EEG might provide richer information and improve staging accuracy, prior studies have demonstrated that single-channel EEG alone can achieve competitive performance. For example, Supratak et al. developed DeepSleepNet,^9^ a deep learning model for automated sleep staging using only single-channel EEG, achieving 82% accuracy on SleepEDF-20, while Eldele et al. introduced AttnSleep,^56^ an attention-based approach for single-channel EEG sleep staging, reaching 84.4% on the same dataset. More recent studies by Lee et al.^14^ and Wang et al.^18^ further validate the efficacy of single-channel EEG in automated sleep scoring. These findings support an EEG-centric approach, though multimodal or multichannel configurations may offer additional benefits in specific clinical or research contexts.

Our work contributes to the growing field of portable sleep monitoring by demonstrating that single-channel EEG can serve as a robust foundation for automated sleep staging. However, future studies could integrate additional physiological signals, such as EOG, to further improve the accuracy and reliability. EOG captures distinct eye movement patterns critical for sleep staging: during N1, it detects slow rolling eye movements and blinks, characteristic of the transition from wakefulness to sleep, while during REM, it detects rapid and irregular eye movements, a hallmark of dreaming activity.^57^ By incorporating EOG data, future iterations of AISleep or similar algorithms could provide deeper insights into sleep physiology, enabling more precise differentiation between sleep stages and improving the overall performance of automated sleep staging systems, especially in portable devices designed for home environments.

### The future application of unsupervised learning algorithms

The combination of UMAP and KDE, with characteristic features serving as weights, represents a promising direction for advancing unsupervised learning algorithms. This approach could be extended beyond sleep staging to other domains within biomedical data analysis, offering a robust framework for unsupervised classification in real-world applications. The potential applications of AISleep are not limited to sleep research; its methodology could inspire new developments in unsupervised learning, particularly in scenarios where interpretability and generalizability are critical.

## Methods

### The sleep datasets

The sleep data were obtained from a publicly available database (SleepEDF^31^^,^^32^) and private sleep recordings from Nanjing Brain Hospital (NJ-EDF dataset). These data contain multiple whole-night polysomnographic sleep recordings, with EEG, EOG, chin EMG, and event markers.

The SleepEDF dataset consists of healthy Caucasian individuals ages 25 to 101 years. Each subject was recorded for two nights, but one night was missing for subjects 13, 36, and 52. The sleep stages were manually scored by well-trained technicians according to the R&K rules.^6^ In this study, we used both SleepEDF-20 (containing 39 nights) and SleepEDF-78 (containing 153 nights) datasets (channel Fpz-Cz). The SleepEDF-20 dataset exclusively consists of sleep data from young individuals, whereas the SleepEDF-78 is an extension of SleepEDF-20, encompassing a wider range of ages by incorporating sleep data from both middle-aged and elderly individuals. To align with the existing AASM rules, S3 and S4 were merged into N3 and excluded sleep frames marked as “MOVEMENT” and “UNKNOWN.”

The NJ-EDF dataset comprises data from 42 patients with sleep disorders, all of whom have specific sleep disorders or self-reported sleep problems. Each subject was recorded for one night. Data use adhered to relevant guidelines and regulations and was approved by the ethics committee. PSG was conducted in accordance with the AASM standards using standard settings and scored by trained and experienced PSG technicians and sleep specialists following AASM guidelines.

### AISleep

We propose AISleep, which utilizes UMAP for dimensionality reduction on the feature space and then employs feature-weighted KDE to infer the distributions of different sleep stages. The overall workflow of AISleep is illustrated in Figure 1B.

#### Preprocessing and feature extraction

For each subject, we extracted a single frontal EEG channel (e.g., Fpz-Cz from SleepEDF dataset) from 30 min before sleep onset to 30 min after sleep offset for unsupervised automatic sleep staging. The EEG signals were sampled at 100 Hz. For every 30-s sleep frame, the PSD was calculated using the Welch method and subsequently converted to the logarithmic unit decibels (dB). To visualize sleep frames in the PSD feature space, we used UMAP, which projects the high-dimensional PSD (within the frequency range of 0.2–30 Hz) into a two-dimensional space, termed PSD_UMAP_.

#### Kernel density estimation

To identify each 30-s sleep frame corresponding to a specific sleep stage, we used KDE^27^ to estimate the probability density function based on a set of PSD_UMAP_ embeddings. The probability density p of a variable x can be estimated using Equation 1:

(Equation 1) $$\begin{array}{c} p\left(x\right)=\frac{1}{nb}\sum_{i=1}^{n}w_{i}K\left(\frac{x-x_{i}}{b}\right)\; \end{array}\text{,}$$

where n is the sample size, $x_{i}$ represents the i-th observation in the sample, K is the kernel function, b is the bandwidth parameter, and $w_{i}$ denotes the weight assigned to $x_{i}$. We chose the Gaussian kernel, as it is one of the most widely used kernels. Its expression is provided below:

(Equation 2) $$\begin{array}{c} K\left(x\right)=\frac{1}{\sqrt{2\pi }}e^{-\frac{x^{2}}{2}} \end{array}\text{.}$$

The bandwidth parameter, b, determines the width of the kernel function and influences the estimation of the density. When the bandwidth is too small, it may cause overfitting, while a larger bandwidth may lead to underfitting. To avoid manual tuning, we used the Scott method for automated bandwidth determination,^28^ as shown in Equation 3:

(Equation 3) $$\begin{array}{c} b=n^{-\frac{1}{4+d_{x}}} \end{array}\text{,}$$

where $d_{x}$ represents the spatial dimension. For two-dimensional space, $d_{x}$ was set to 2.

In this study, $x_{i}$ refers to the PSD_UMAP_ embedding at each 30-s sleep frame. The weights $w_{i}$ for different sleep stages were determined based on the characteristic signals and features of different sleep stages, such as gamma power, fast spindle, SOs, and PSD_osc_ (the oscillatory component of PSD), which will be introduced in the following sections.

#### Gamma power

To discern Wake_open_, characterized by elevated gamma power, we employed KDE to estimate the probability density function for PSD_UMAP_. The KDE weights $w_{i}$ were dynamically assigned based on gamma power, with the Wake_open_ estimation detailed in Algorithm 1.

Algorithm 1 **The Wakeopen estimation**

**Input:** PSD**,** Power Spectrum Density**;**

 ${PSD}_{UMAP}$, UMAP embeddings of PSD**.**

**Output:** S, Sleep stages (including 0: Wake, 5: Unknown).

1: Initialize all stages (5) in S as Unknown (5)
2: ${PSD}_{gamma}=MeanPower$ (PSD,band=(25,50))
3: th = Otsu^34^ (${PSD}_{gamma}$)
4: $\mu_{high}$ = Mean (${PSD}_{gamma}\left[{PSD}_{gamma}>th\right]$)
5: For i in $Range\left(Len\left({PSD}_{gamma}\right)\right)$:
6:  If ${PSD}_{gamma}\left[i\right]$ > $\mu_{high}$:
7:  $w_{i}$ = 1
8:  Elif ${PSD}_{gamma}\left[i\right]$ < th:
9:  $w_{i}$ = 0
10:  Else:
11:  $p_{rank}=PercentileRank({PSD}_{gamma}\left[{PSD}_{gamma}>th\right],{PSD}_{gamma}\left[i\right]$)
12:  $w_{i}$ = 2 ∗ p_rank
13: p = $KDE({PSD}_{UMAP}$, w)
14: S[p<max(p)∗0.1] = 0 # Wake
15: Return S

#### Fast spindle power

To identify the N2 and primarily N3 stage (denoted as N2N3), characterized by *P*_*sp*_, we employed KDE to estimate the probability density function based on a set of PSD_UMAP_ embeddings. The KDE weights $w_{i}$ were dynamically determined by *P*_*sp*_, with the N2N3 estimation detailed in Algorithm 2.

Algorithm 2 **The N2N3 estimation**

**Input:** PSD, Power Spectrum Density;

 ${PSD}_{UMAP}$, UMAP embeddings of PSD;

 S, Sleep stages (including 0: Wake, 5: Unknown).

**Output:** S, Sleep stages (including 0: Wake, 2: N2N3, 5: Unknown).

1: ${PSD}_{osc}$, ${PSD}_{fra}$ = IRASA(PSD)
2: ${PSD}_{osc}$ = GaussianFilter2D (${PSD}_{osc}$)
3: For i in $Range\left(Len\left({PSD}_{osc}\right)\right)$:
4:  $f_{i}$ = $\mathrm{ArgMax}\left({PSD}_{osc}\left[i\right]\right)$
5: $f_{peak}$ = FindPeak⁡(KDE(f),14) # find peak frequency closest to 14Hz
6: $P_{sp}$ = $MeanPower\left(PSD,band=\left(f_{peak}-1,f_{peak}+1\right)\right)$
7: $\mu_{N2N3},\sigma_{N2N3}=GaussianFit\;\left(P_{sp}\left[P_{sp}\geq 0\right]\right)$
8: $\mu_{non-N2N3},\sigma_{non-N2N3}=GaussianFit\;\left(P_{sp}\left[P_{sp}<0\right]\right)$
9: $w_{N2N3}=F\left(P_{sp};\mu_{N2N3},\sigma_{N2N3}\right)$
10: $w_{non-N2N3}=1-F\left(P_{sp};\mu_{non-N2N3},\sigma_{non-N2N3}\right)$
11: $p_{N2N3}=KDE\left({PSD}_{UMAP},weight=w_{N2N3}\right)$
12: $p_{non-N2N3}=KDE\left({PSD}_{UMAP},weight=w_{non-N2N3}\right)$
13: $S\left[p_{N2N3}>p_{non-N2N3}\right]=2$ #N2N3
14: Return S

We first applied IRASA^35^ to decompose the PSD into two components as described in Algorithm 2. In EEG signals, the fractal component ${\text{PSD}}_{\text{fra}}$ usually indicates background brain electrical noise, commonly known as pink 1/f noise in biological systems,^58^ while ${\text{PSD}}_{\text{osc}}$ represents the oscillatory components of the PSD, such as spindle and alpha waves.

Based on the cumulative density function derived from the Gaussian distribution (Equation 4), weights ($w_{N2N3}$ and $w_{∼N2N3}$) in Algorithm 2 for each embedding were calculated as follows:

(Equation 4) $$\begin{array}{c} F\left(x;\mu ,\sigma \right)=\frac{1}{\sigma \sqrt{2\pi }}\int_{-\infty }^{x}\;\exp\left(-\frac{{\left(x-\mu \right)}^{2}}{2\sigma^{2}}\right)dx \end{array}\text{.}$$

#### The slow oscillation percentage

To identify the N3 stage, characterized by SOs, we used KDE to estimate the probability density function based on PSD_UMAP_ embeddings. The KDE weights $w_{i}$ were dynamically determined by the SO, with the N3 estimation detailed in Algorithm 3.

Algorithm 3 **The N3 estimation**

**Input:** PSD, Power Spectrum Density**;**

 ${PSD}_{UMAP}$, UMAP embeddings of PSD;

 ${SO}_{durations}$, the duration of slow waves in each sleep frame;

 ${frame}_{durations}$, 30 s;

 S, Sleep stages (including 0: Wake, 2: N2N3, 5: Unknown).

**Output:** S, Sleep stages (including 0: Wake, 2: N2, 3: N3, 5: Unknown).

1: ${SO}_{percentage}={SO}_{durations}/{frame}_{durations}$
2: $w=\max\left({SO}_{percentage}-10\%,0\right)$
3: $p_{N3}$ = KDE (${PSD}_{UMAP}$, w)
4: ${N3}_{candidates}$ = $\mathrm{Arg}\left(p_{N3}<\mathrm{Max}\left(p_{N3}\right)*0.1\right)$
5: For i in ${N3}_{candidates}$
6:  ${PSD}_{UMAP}^{Wake}={PSD}_{UMAP}\left[S==0\right]$
7:  ${PSD}_{UMAP}^{N2N3}={PSD}_{UMAP}\left[S==2\right]$
8:  If $distance({PSD}_{UMAP}\left[i\right]$, ${PSD}_{UMAP}^{N2N3}$) < distance (${PSD}_{UMAP}\left[i\right]$, ${PSD}_{UMAP}^{Wake}$):
9:  S[i]=3 # N3
10:  Else:
11:  S[i]=0 # Wake
12: Return S

We used the SO detection algorithm developed by YASA,^13^ which was adapted from previous studies,^57^^,^^59^ using the following criteria:

(1) a frequency range of 0.5–2.0 Hz,
(2) peak-to-peak amplitude >75 μV,
(3) duration of the negative deflection >300 ms and <1,500 ms,
(4) duration of the positive deflection >100 ms and <1,000 ms,
(5) negative peak < −10 μV and positive peak >10 μV.

#### The strength of oscillatory activities

During sleep, the primary oscillations within the frequency range of 5–20 Hz are spindle and alpha rhythms. To identify the Wake_close_ stage, characterized by alpha rhythms, we first needed to identify the sleep frames exhibiting the oscillatory activity. We used KDE to estimate the probability density of the oscillatory activities based on PSD_UMAP_ embeddings. The KDE weights were dynamically assigned according to ${PSD}_{osc}$, with the Wake_close_ estimation detailed in Algorithm 4.

Algorithm 4 **The Wakeclose estimation**

**Input:** ${PSD}_{osc}$**,** the oscillatory component of PSD**;**

 ${PSD}_{UMAP}$**,** embeddings of PSD;

 S, Sleep stages (including 0: Wake, 2: N2, 3: N3, 5: Unknown).

**Output:** S, Sleep stages (including 0: Wake, 2: N2, 3: N3, 5: Unknown).

1: ${Std}_{osc}$ = std (${PSD}_{osc}$ [5<freqs <20, : ], axis=1)
2: ${Std}_{overall}$ = std (${PSD}_{osc}$)
3: $\mu_{osc},\sigma_{osc}$ = GaussianFit (${Std}_{osc}$ [${Std}_{osc}$ ≥${Std}_{overall}$])
4: $\mu_{non-osc},\sigma_{non-osc}=GaussianFit\left({Std}_{osc}\left[{Std}_{osc}<{Std}_{overall}\right]\right)$
5: $w_{osc}=F\left({Std}_{osc};\mu_{osc},\sigma_{osc}\right)$
6: $w_{non-osc}=1-F\left({Std}_{osc};\mu_{non-osc},\sigma_{non-osc}\right)$
7: $p_{osc}=KDE\left({PSD}_{UMAP},weight=w_{osc}\right)$
8: $p_{non-N2N3}=KDE\left({PSD}_{UMAP},weight=w_{non-osc}\right)$
9: $S\left[p_{N2N3}>p_{non-N2N3}\right]=2$ # N2N3
10: ${Wake}_{\text{candidates}}$ = $\mathrm{Arg}\left(p_{osc}>p_{non-N2N3}\right)$
11: For i in ${Wake}_{\text{candidates}}$
12:  If S[i]==5:
13:  S[i]=0 # ${Wake}_{close}$
14: Return S

#### The estimation of N1 and REM

The Wake, N2, and N3 stages were identified from the preceding steps. However, distinguishing between the N1 and the REM stages remains a challenge because they both exhibit low-amplitude mixed frequency (LAMF) signals and highly similar PSD.

Typically, the N1 stage is brief, lasting between 1 and 5 min, whereas REM sleep is longer and increases in duration with total sleep time. The initial REM period lasts approximately 10 min, while the final one can last up to an hour.^60^ Moreover, transitions between stages are not uniform: the transition from Wake to N1 is quite common, while the likelihood of transiting from N2 to REM is notably higher.^61^ Based on these previous findings, we designed the algorithm presented in Algorithm 5 to distinguish N1 from REM.

Algorithm 5 **The estimation of N1 and REM**

**Input:** S, Sleep stages (including 0: Wake, 2: N2, 3: N3, 5: Unknown);

 kernelsize, Length of the convolution kernel (set to 20, representing a 10-minute window size).

**Output:** S, Sleep stages (including all sleep stages).

1: Initialize all Unknown stages (5) in S as REM (4)
2: $S_{smoothed}$ = ConvolveSmoothed(S,kernelsize)
3: For t in $Range\left(Len\left(S_{smoothed}\right)\right)$:
4:  If S[t]==4 and Duration(S[t]) < 10 minutes:
5:  If ($S_{smoothed}\left[t\right]-1$) < ($4-S_{smoothed}\left[t\right]$):
6:  S[t]=1 # N1
7:  $S_{smoothed}$ = ConvolveSmoothed(S,kernelsize)
8: Return S

## Evaluation metrics

We compared AISleep’s performance with two traditional unsupervised methods: *k*-means and GMM. To evaluate the effectiveness of unsupervised clustering for sleep staging, we applied a modified Jonker-Volgenant algorithm^62^ to optimize the alignment between unsupervised cluster outputs and the true sleep stage labels, ensuring an objective assessment of accuracy.

To evaluate the performance of AISleep, we first computed the normalized confusion matrix, with each entry [*i, j*] indicating the proportion of frames actually belonging to sleep stage *i* that were predicted as stage *j*. Based on the confusion matrix, we then computed multiple performance metrics:

(1) Per-class F1 scores: the harmonic mean of precision and recall for each sleep stage individually:

(Equation 5) $$Precision=\frac{TP}{TP+FP}$$

(Equation 6) $$Recall=Sensitivity=\frac{TP}{TP+FN}$$

(Equation 7) $$F1=\frac{2\times \left(Precision\times Recall\right)}{\left(Precision+Recall\right)}$$

where TP, FP, and FN denote true positives, false positives, and false negatives, respectively.

(2) Overall metrics

Accuracy (ACC): the proportion of correctly predicted sleep frames out of the total sleep frames.

Macro F1 score (MF1): the mean of the per-class F1 scores, ensuring balanced evaluation across all sleep stages.

Cohen’s kappa (κ): quantifies the agreement between the model’s predictions and the expert annotations, accounting for chance consistency.

### Cross-domain testing

To evaluate the performance of the unsupervised AISleep algorithm relative to supervised methods on unseen datasets, we conducted cross-domain testing. Since YASA has been trained across seven distinct sleep datasets and offers open source software tools, we were able to directly assess the performance of YASA on the unseen datasets. In this cross-domain testing, supervised methods, such as TinySleepNet and SleePyCo, were trained on one dataset and then evaluated on another. For healthy subjects, TinySleepNet and SleePyCo were trained on the SleepEDF-78 and then evaluated on the NJ-EDF. For patients with sleep disorders, TinySleepNet and SleePyCo were trained on the SleepEDF-78 and then evaluated on the NJ-EDF dataset. The performance metrics for supervised methods were derived from the average results of 5-fold cross-validation models.

### Statistical analysis

We employed the Shapiro-Wilk test to assess the normality of the distribution of differences in paired sample data. If these differences were normally distributed, a paired sample t test was conducted to compare the mean differences; otherwise, the non-parametric Wilcoxon signed-rank test was applied. For independent sample data, if the distribution was normal, a two-sample t test was used to compare the means of the two groups; if not, the non-parametric Mann-Whitney U test was employed.

For comparisons involving more than two groups, we used one-way ANOVA when the data were normally distributed or the non-parametric Kruskal-Wallis H test when the data did not follow a normal distribution, provided the sample size was sufficiently large. We adjusted *p* values using the Holm method, a step-down procedure that applies Bonferroni adjustments to control the family-wise error rate in multiple testing scenarios.

## Resource availability

### Lead contact

Requests for further information and resources should be directed to, and will be fulfilled by, the lead contact, Yina Wei (weiyina@fudan.edu.cn).

### Materials availability

This study did not generate new unique reagents.

### Data and code availability

The sleep data were obtained from the SleepEDF database^31^^,^^32^: https://doi.org/10.13026/C2X676. The NJ-EDF dataset is available from Nanjing Brain Hospital Affiliated to Nanjing Medical University, but restrictions apply to the availability of these data, which were used under license for the current study and are therefore not publicly available. However, data are available from the authors upon reasonable request and with the permission of Nanjing Brain Hospital Affiliated to Nanjing Medical University. Our source code is available at GitHub: https://github.com/mx597014232/AISleep and has been archived at Zenodo: https://doi.org/10.5281/zenodo.16874797.^63^

## Acknowledgments

The New Cornerstone Science Laboratory, Institute for Brain and Intelligence, Fudan University is Y.W.’s primary affiliation; all others are secondary affiliations for this author. This work was supported by the 10.13039/501100001809National Natural Science Foundation of China (32471148), the 10.13039/100022963Key Research and Development Program of Zhejiang Province (2024C01142), and Scientific Projects of Zhejiang Lab & Shanghai Artificial Intelligence Laboratory (K2023KA1BB01), China.

## Author contributions

Conceptualization, X. Mai, Y.W., L.Z., and H.P.; methodology, X. Mai and Y.W.; investigation, X. Mai, Y.W., L.Z., and H.P.; data curation, J.Z. and X.J.; formal analysis and visualization, X. Mai; writing – original draft, X. Mai and Y.W.; writing – review & editing, all authors; supervision, project administration, and funding acquisition, Y.W.; resources, Y.W.

## Declaration of interests

The authors declare no competing interests.

## Supplemental information

Document S1. Figures S1–S3 and Table S1

Document S2. Article plus supplemental information
