# Supplementary material for: AISleep: Automated and interpretable sleep staging from single-channel EEG data
Source: Patterns (N Y). 2025 Sep 24;6(12):101367. doi: 10.1016/j.patter.2025.101367 (PMC12745993; doi:10.1016/j.patter.2025.101367)
Supplement: Document S1. Figures S1–S3 and Table S1 [file mmc1.pdf]

Patterns, Volume *6*

Supplemental information

AISleep: Automated and interpretable sleep staging from single-channel EEG data

Xun Mai, Binghua Song, Manli Luo, Jun Zhu, Xu Jiang, Xiao Ma, Feng Lin, Xiaoqing Hu, Hanchuan Peng, Li Zhang, and Yina Wei

SUPPLEMENTAL INFORMATION

Figures S1–S3, and Tables S1.

Supplemental tables

Table S1.

Sleep Stage		EEG Spectral Features	Waveform Features
Wake	open eye	This stage is characterized by elevated power across all frequency bands. Notably, gamma power (25-100 Hz), which is linked to cognitive processes, is particularly prominent during this state.	Various eye movement waveforms
	close eye	This stage is characterized by decreased gamma power and increased alpha (8-13 Hz) power. Approximately 10% of people do not exhibit alpha rhythms when their eyes are closed.	Continuous sinusoidal alpha rhythm
N1		This stage is characterized by a decrease in power across all frequency bands, with brain activity predominantly consisting of low-amplitude mixed-frequency (LAMF, 4-7 Hz) signals.	Slow eye movements; Vertex waves (V-waves)
N2		This stage is characterized by an increase in delta wave (0-4 Hz) and the presence of sleep spindles (12-16 Hz).	K-complex; Spindle
N3		As sleep deepens, delta wave (0-4 Hz) increases, while sleep spindle activity (12-16 Hz) decreases.	Slow waves
REM		This stage is very similar to N1, characterized by lower power across all frequency bands and dominated by LAMF (4-7 Hz) brain activity.	Rapid eye movements; Sawtooth waves (2-6 Hz)

Table S1. Summary of the EEG characteristics of different sleep stages.

Supplemental figures

Figure S1

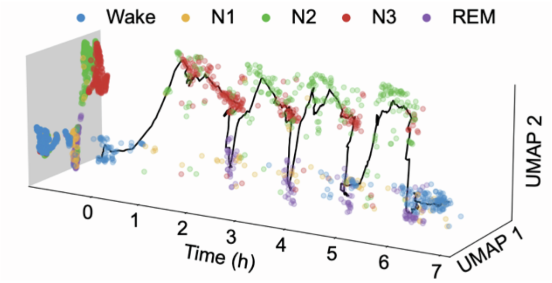

Figure S1. Trajectory of EEG embeddings (PSD$_{UMAP}$) in feature space across a full night of sleep (Subject: SC4001). Each dot represents a 30-second sleep frame, colored by expert-annotated sleep stages. The black curve indicates the moving average trajectory of sleep stages throughout the night.

Figure S2.

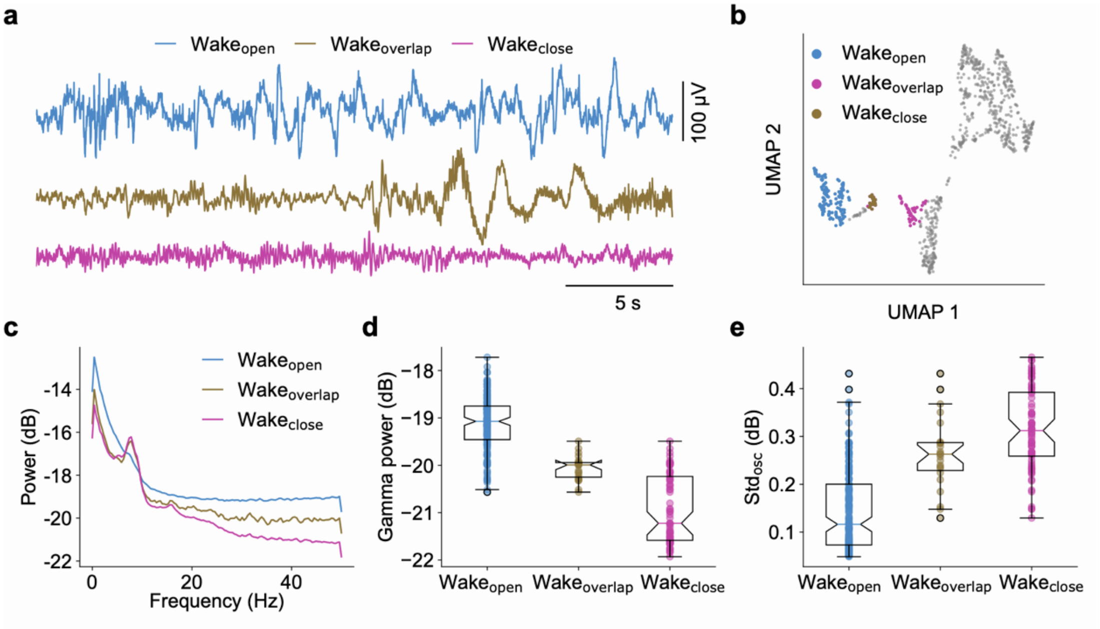

Figure S2. EEG activity during the Wake stage.
(a) Examples of EEG activity during three Wake substates: Wake$_{open}$ (characterized by gamma activity), Wake$_{overlap}$ (characterized by both alpha and gamma activity), and Wake$_{close}$ (characterized by alpha activity).
(b) PSD$_{UMAP}$ projection of each sleep frame across one night of sleep. Colored dots represent the Wake stage, while gray dots denote other sleep stages.
(c) PSDs of Wake substates.
(d) Gamma power across Wake substates.
(e) Std$_{osc}$ (strength of oscillatory activity, e.g., alpha) across Wake substates.

Figure S3.

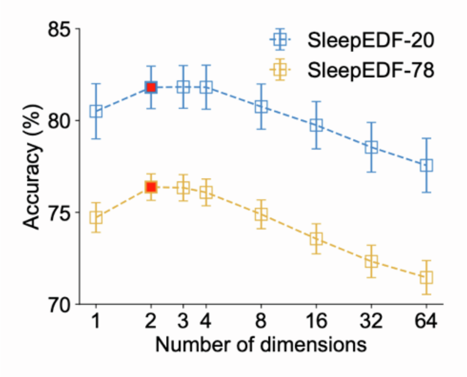

Figure S3 Sleep staging performance varies with UMAP embedding dimensions in healthy subjects. The red square highlights the optimal performance.
